# Impact of E-Cigarette Use on Circadian Proteins and Cardiovascular Risk Markers

**DOI:** 10.1007/s12265-026-10762-y

**Published:** 2026-03-09

**Authors:** Ayman Alzu’bi, Ejlal Abu-El-Rub, Ramada R. Khaswaneh, Mohammed Al-Zubaidi, Wissam Almomani, Abdelrahman Alenaizat, Adnan H. Ayyash, Anas Alragheb, Anas Alshannag, Enas Ahmad, Mai Elaarag, Raed M. Al-Zoubi

**Affiliations:** 1 Department of Basic Medical Sciences, Faculty of Medicine, Yarmouk University, Irbid, 211-63 Jordan; 2 Surgical Research Section, Department of Surgery, Hamad Medical Corporation, Doha, Qatar; 3 Department of Biomedical Sciences, QU-Health, College of Health Sciences, Qatar University, 2713 Doha, Qatar; 4 Department of Chemistry, Jordan University of Science and Technology, P.O.Box 3030, Irbid, 22110 Jordan

**Keywords:** E-cigarette, Vaping, Circadian system, Melatonin, BMAL1

## Abstract

The increasing popularity of E-cigarettes among young adults has raised concerns about their health effects, particularly on the circadian system, which regulates critical physiological processes. This study examined how vaping influences circadian proteins and inflammatory markers, comparing these effects to those in cardiovascular disease patients. Blood samples from 254 participants, non-vaping controls (n = 90), regular vapers (n = 86), and cardiovascular patients (n = 78), were analyzed for circadian proteins (Melatonin, BMAL1, PER1, PER2, CRY1, CRY2) and inflammatory/oxidative stress markers (IFN-γ, MDA). Vaping significantly decreased Melatonin, PER1, PER2, CRY1, and CRY2, while BMAL1 remained unchanged. Elevated IFN-γ and MDA levels indicated increased inflammation and oxidative stress in vapers. Vaping induces circadian disruption patterns similar to cardiovascular disease, suggesting a potential mechanism linking e-cigarette use to increased cardiovascular risk.

**Graphical Abstract:**

**Figure:**
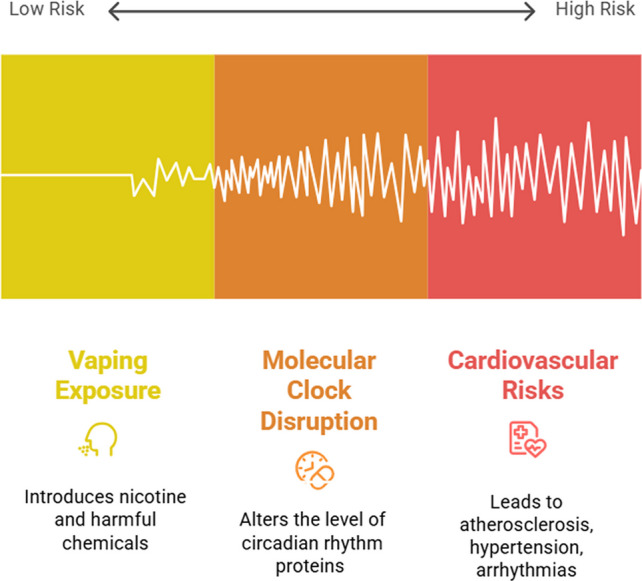
The figure illustrates a progression from vaping exposure toincreased cardiovascular risk. Vaping introduces nicotine and harmful chemicals (low risk stage), leading todisruption of circadian clock proteins (reduced melatonin, PER1/2, CRY1/2; unchanged BMAL1) and elevatedinfl ammatory and oxidative stress markers (IFN-γ, MDA). These molecular alterations resemble patterns observed incardiovascular disease, suggesting a mechanistic link between e-cigarette use and heightened cardiovascular risk.

## Introduction

The use of E-cigarettes or vaping has increased rapidly worldwide, particularly among adolescents and young adults [1]. The surge in utilizing E-cigarettes is due to its perceived safety compared to traditional tobacco-based cigarettes and hookah, as people inhale only the nicotine vapor without tobacco [2]. E-cigarette or vaping has been initially implemented to assist those who are considered tobacco addicted in smoking cessation [3]. Several studies reported controversial results regarding the long-run effects of E-cigarette or vaping on various body systems and regulatory pathways [2, 4, 5].

Circadian rhythm or system is an endogenous system regulated by the hypothalamus and the melatonin hormone to generate the 24-h biological clock, which synchronizes with the environmental daylight cycle and plays a pivotal role in controlling various cellular signaling pathways to ensure optimal physiological functions [6]. Several reports highlighted the importance of maintaining proper circadian rhythm, and any disruption or misalignment increases the risk of developing pathological changes and triggers serious illnesses [7, 8]. The circadian system includes various proteins that participate in controlling various biological processes and adjusting their function according to the body's needs [9]. These circadian proteins are crucial for modulating metabolism, survival, inflammation, apoptosis, proliferation, and autophagy, which are essential for cellular homeostasis [10]. The oscillation in the level of various circadian proteins depends on the level of two master regulators: Melatonin and Brain and Muscle Arnt-like (BMAL1) [10]. Hypothalamus signals the pineal gland to initiate the synthesis of Melatonin, which is integral for circadian system regulation [11]. Melatonin, in turn, increases the level of an important circadian protein, the BMAL1 [12]. BMAL1 forms a complex with another circadian protein, the CLOCK to stimulate the transcription of two groups of clock proteins; the Period proteins, which encompass three proteins, PER1, PER2, and PER3, and the Cryptochrome proteins, which comprise two proteins, CRY1 and CRY2 [13]. The formed CRY and PER proteins translocate to the nucleus and induce an inhibitory effect to suppress the activity of the BMAL1-CLOCK complex [14]. Based on that, BMAL1-CLOCK acts as an enhancer (positive pole) while CRY and PER are considered the suppressors (negative pole) of the circadian system feedback loop that is crucial for regulating the biological clock rhythmicity and functionality.

CRY proteins instigate a potent inhibitory effect on the activity of BMAL1-CLOCK complex compared to a mild one initiated by PER proteins [10]. The correlation between vaping and circadian dysregulation has not been thoroughly investigated. One study reported that E-cigarette vapor exposure can alter the level of Clock genes in the lung and negatively impact its metabolism and their defensive abilities against bacterial infections [15]. Scarcity in data about the systemic effects of vaping must be taken into consideration and carefully examined.

These circadian proteins are critically involved in cardiovascular regulation: BMAL1 and CLOCK modulate endothelial function, blood pressure, and heart rate variability, while PER and CRY protect the heart from oxidative stress, inflammation, and fibrosis. Disruption of these protein levels can lead to autonomic dysfunction, pro-inflammatory cytokine release, and increased cardiovascular risk [16, 17]. Given the central role of circadian proteins in cardiovascular homeostasis, this study aims to investigate the impact of E-cigarette use on circadian protein levels and inflammatory cytokines, providing insight into possible cardiovascular implications.

## Method

### Ethical Approval and Consent to Participate

This study was approved by the Institutional Review Board at Yarmouk University (IRB/2023/536/). Written informed consent was obtained from all participants prior to sample collection.

### Study Participants

A total of 254 participants were recruited for this study. Written informed consent was obtained from all participants. All participants were requested to fill out questionnaires for socio-demographic variables, including gender, age, history of using E-cigarettes and traditional smoke, number of sleeping hours/day, history of cardiovascular diseases or any other chronic illness, and history of regular prescriptions. Participants were instructed to refrain from vaping for at least 8 h prior to sample collection to minimize acute effects. Blood samples were collected from all participants via venipuncture between 9–11 a.m. to minimize circadian variation, centrifuged at 1500 × g for 15 min at 4 °C, aliquoted, and stored at − 80 °C within one hour of collection.

The collected blood samples were divided into three groups: 1) The non-vaping (control, n = 90) group, which included healthy adults who have never used E-cigarettes, traditional smoke, or any other tobacco products. 2) The vaping group (n = 86) included healthy individuals who were regular users of E-cigarettes (15–20 time/day, each consisting of around 15–20 puffs) for at least one year and not users of any other tobacco products. 3) The cardiovascular group (n = 78) included individuals with a history of cardiovascular diseases (including hypertension and heart failure) and no history of using E-cigarettes or any other tobacco products.

Exclusion criteria for the three groups also included shift workers, individuals with daytime sleep schedules, and individuals with insomnia disorder, neurological disorders, psychiatric disorders, chronic illness, and a history of alcohol and drug abuse.

The study groups (non-vaping controls, vaping participants, and cardiovascular patients) were not formally matched. Demographic characteristics including age, sex, and sleep duration were recorded and reported descriptively (Table 1). No formal statistical adjustment or matching was performed due to limited covariate data, such as BMI, physical activity, or diet.

**Table 1:** Demographic and clinical characteristics of participants

Group	n (Male/Female)	Mean Age (years)	History of Cardiovascular Diseases (% of participants)	Average Sleep Duration (% of participants)
Non-vaping Control	90 (48/42)	41.67	-	< 6 h/day (2.2%) 6–10 h/day (97.8%) > 10 h/day (0%)
Vaping Group	86 (47/39)	40.41	-	< 6 h/day (4.7%) 6–10 h/day (95.3%) > 10 h/day (0%)
Cardiovascular Group	78 (45/33)	45.6	hypertension (57.4%) heart failure (42.6%)	< 6 h/day (3.8%) 6–10 h/day (93,6%) > 10 h/day (2.6%)

### Elisa Assays

ELISA assays were performed to detect the serum levels of Melatonin (ELK Biotechnology, Cat: ELK9164), BMAL1 (ELK Biotechnology, Cat: ELK5308), PER1 (ELK Biotechnology, Cat: ELK6571), PER2 (ELK Biotechnology, Cat: ELK9429), CRY1 (ELK Biotechnology, Cat: ELK3896), CRY2 (BT LAB, Cat: E5127Hu), and IFN-γ (ELK Biotechnology, Cat. ELK1036). The assays procedures were performed following the manufacturers’ instructions. The intra- and inter-assay coefficient of variation (CV%) for all ELISA kits ranged between 4.5–8.2% and 6.1–9.5%, respectively, according to manufacturer specifications (ELK Biotechnology, BT LAB, R&D Systems).

### Assessment of Malondialdehyde (MDA) Levels

The mean serum level of MDA, as a marker of lipid peroxidation, was measured using a commercially available TBARS assay kit (R&D Systems, KGE013) and normalized to protein concentration. The assay procedure was performed following the manufacturer’s instructions. The colored product of the reaction of MDA with thiobarbituric acid was measured spectrophotometrically at 532 nm. The MDA content was expressed as nmol/mg protein.

### Statistical Analysis

Statistical analyses were performed using GraphPad Prism (version 8.0.0) and verified in SPSS (version 25). Data are presented as mean ± SEM. Data distribution was tested using the Shapiro–Wilk test, and homogeneity of variances was checked with Levene’s and Brown–Forsythe tests. When these assumptions were met, group comparisons were conducted using one-way ANOVA followed by Tukey’s post hoc test. If assumptions were violated, analyses were performed using Welch’s ANOVA with Games–Howell post hoc test or the Kruskal–Wallis test with Dunn–Holm correction, as appropriate. To reduce the risk of false-positive results due to multiple comparisons, p-values were adjusted using the Benjamini–Hochberg false discovery rate (FDR) method across the family of primary biomarkers. A two-tailed nominal α = 0.05 was used to determine statistical significance.

For the measured biomarkers, Shapiro–Wilk tests indicated that Melatonin, BMAL1, PER2, CRY1, and IFN-γ levels were normally distributed across all groups, while PER1 and CRY2 levels violated normality in the cardiovascular group. Levene’s test revealed that homogeneity of variance was met for all markers except MDA. Accordingly, one-way ANOVA with Tukey’s post hoc was applied to comparisons meeting assumptions, Welch’s ANOVA with Games–Howell post hoc was used for MDA, and Kruskal–Wallis with Dunn–Holm correction was used for PER1 and CRY2.

Additionally, pairwise Spearman correlation coefficients were calculated among the six circadian proteins, IFN-γ, and MDA to assess inter-marker relationships and potential covariation.

## Results

### Sample Characteristics

As shown in Table 1, ninety participants (48 males and 42 females) with a mean age of 41.67 years (age range: 35–50 years) were recruited in the control group. The number of sleeping hours for the majority (97.8%) of the participants in this group were 6–10 h/day.

Eighty-six participants (47 males and 39 females) with mean age of 40.41 years (age range: 35–47 years) were recruited in the vaping group. 11.6% of the participants reported they have been using e-cigarette products for 1–2 years and 88.4% reported using e-cigarette products for more than 2 years. The majority (95.3%) reported 6–10 sleeping hours/day, 4.7% of the participants reported having sleeping hours less than 6 h/day (Table 1).

Seventy-eight participants (45 males and 33 females) with a mean age of 45.6 years (age range: 37–50 years) were recruited in the cardiovascular group. 57.4% were hypertensive, and 42.6% were diagnosed with heart failure. The number of sleeping hours for the majority (93,6%) of the participants in this group were 6–10 h/day. 3.8% reported having sleeping hours less than 6 h/day, and 2.6% reported having more than 10 sleeping hours/day. The majority (82.6%) of the participants reported they are on regular prescriptions (Table 1).

## Vaping Disrupts the Master Regulators of the Circadian System

**Fig. 1:**
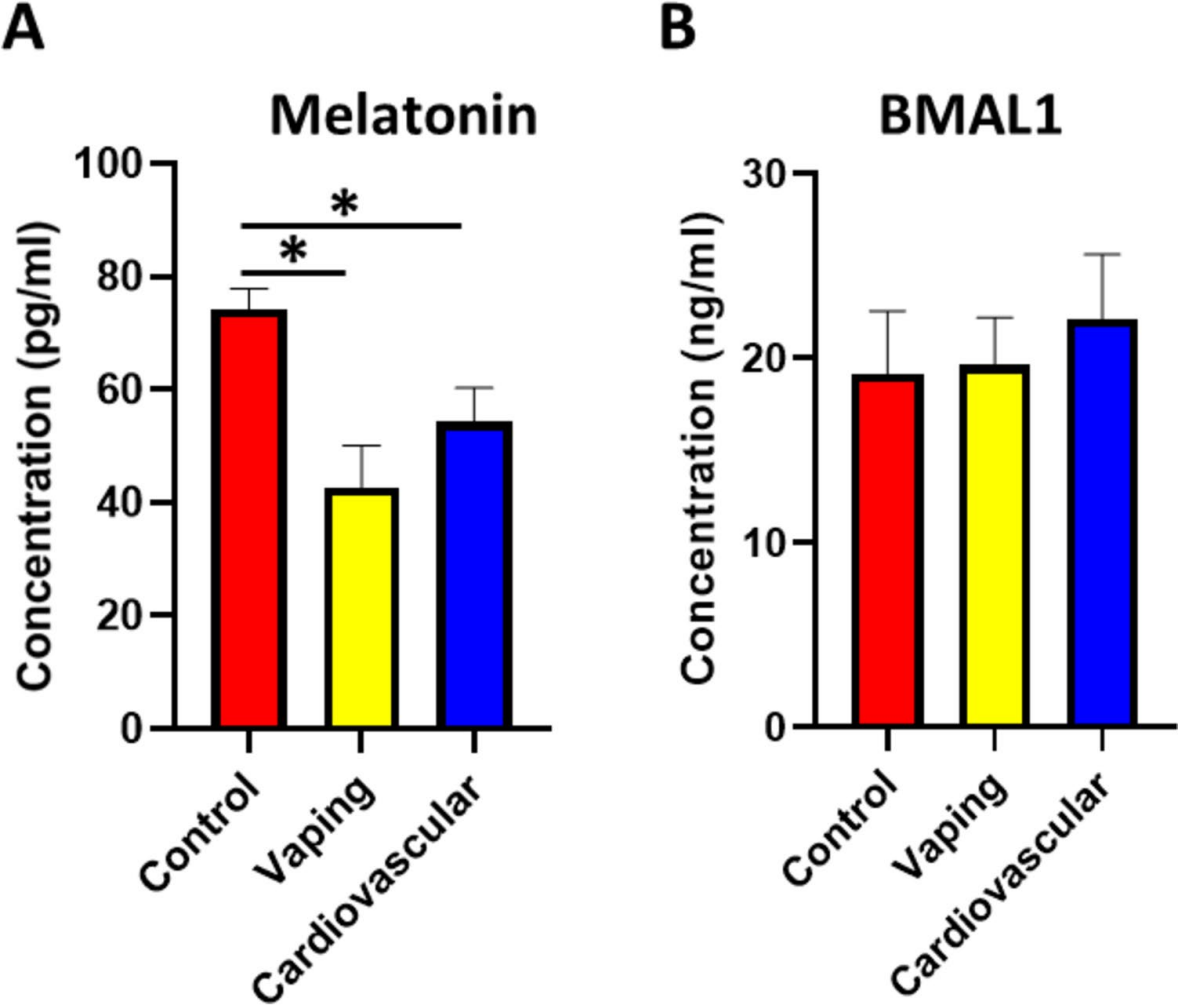
The serum levels of Melatonin and BMAL1. **A** The serum Melatonin levels were significantly reduced in both the vaping (F(2,251) = 15.32, *p* = 0.00003, η^2^ = 0.11, 95% CI [0.06, 0.16]; vaping vs. control p = 0.00003) and cardiovascular (*p* = 0.00005 vs. control) groups compared to controls. **B** The serum BMAL1 levels did not differ significantly among groups (F(2,251) = 1.87, *p* = 0.16, η^2^ = 0.01, 95% CI [0.00, 0.04]). Data presented as mean ± SEM; *p < 0.05. The number of participants in the Control group, Vaping group, and Cardiovascular group are 90, 86,78 respectively

To determine if vaping disrupts the master circadian regulators, we examined the levels of key circadian markers, melatonin and BMAL1, in the serum of vaping and control samples. Melatonin sits at the top of the hierarchy for regulating the circadian system. This hormone regulates the sleep–wake cycle and initiates the synthesis of other circadian proteins, particularly BMAL1 [18]. By comparing melatonin levels in the vaping vs non-vaping control group, we observed a significant decrease in the level of melatonin in the serum of vaping samples (Fig. 1A).

The disturbance in the circadian system markers is reported to be a risk factor for cardiovascular diseases (CVDs) by inducing chronic stress and modulating the functionality of the cardiovascular system. We compared the reduction in melatonin level in the vaping group with the level of melatonin detected in the cardiovascular non-vaping group. The results showed a significant decrease in melatonin level in the cardiovascular non-vaping group, and the reduction was comparable to the one detected in the vaping group (Fig. 1A), indicating that vaping may pose a risk to cardiovascular disorders.

We further investigated the level of the core clock protein BMAL1, which plays a vital role in regulating the level of downstream circadian markers, including PERs and CRYs, and considered the positive pole of the circadian system feedback loop [19]. The results indicated no significant difference in BMAL1 level between the control, vaping, and cardiovascular non-vaping groups (Fig. 1B).

## The Effect of Vaping on Period Circadian Proteins (PER1 and PER2)

**Fig. 2:**
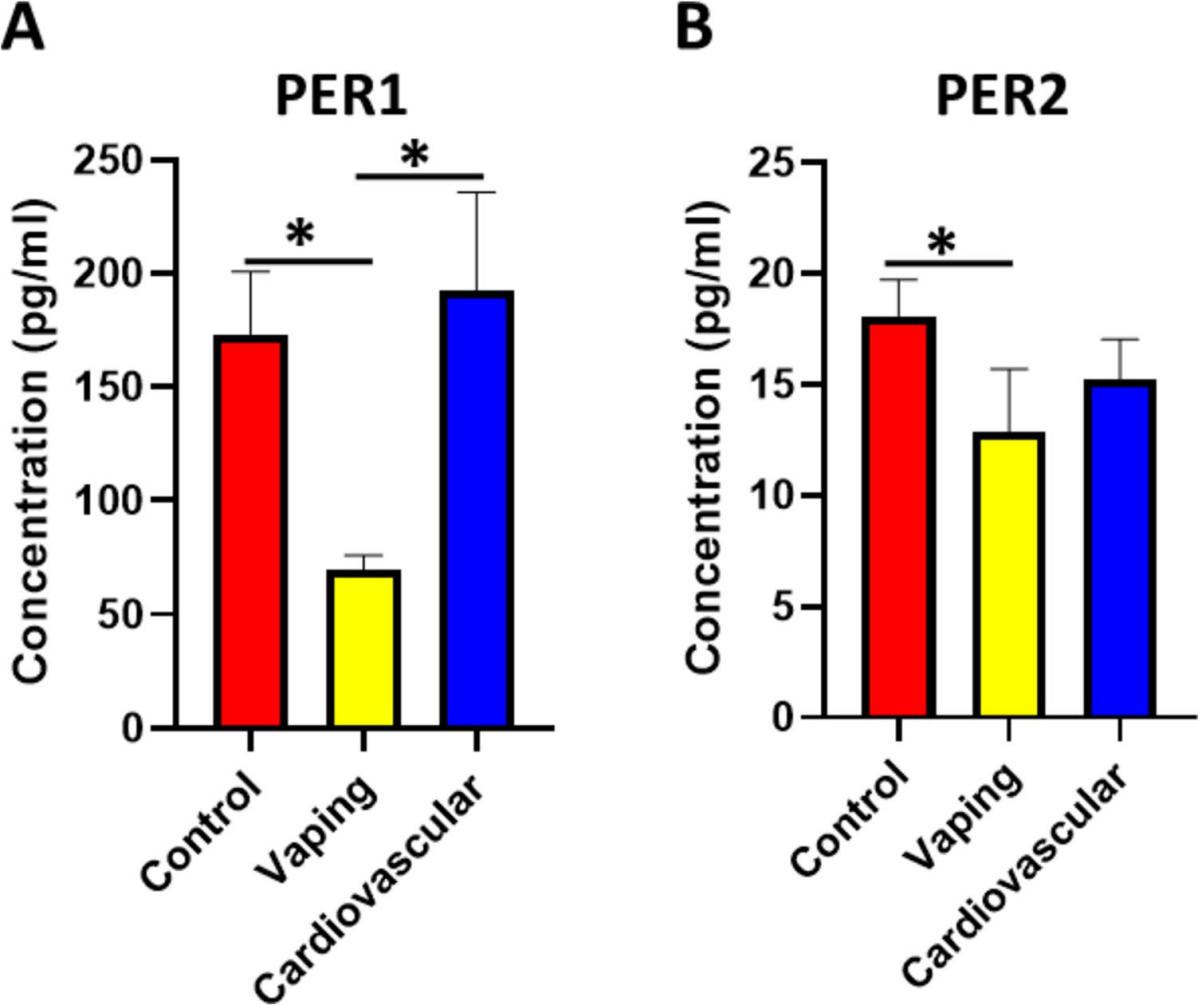
The serum levels of PER1 and PER2. **A** The serum PER1 levels differed significantly among groups (Kruskal–Wallis H(2) = 21.45, *p* < 0.001, r = 0.29). Post hoc tests showed lower PER1 in the vaping group vs. controls (p = 0.002) and higher PER1 in cardiovascular group vs. controls (*p* = 0.04). **B** The serum PER2 levels were analyzed by one-way ANOVA (F(2,251) = 12.08, *p* = 0.00006, η^2^ = 0.09, 95% CI [0.04, 0.14]); PER2 was significantly lower in the vaping group compared to controls (*p* < 0.001), with no significant difference in cardiovascular patients (*p* = 0.18). Data presented as mean ± SEM; *p < 0.05. The number of participants in the Control group, Vaping group, and Cardiovascular group are 90, 86,78 respectively

Period circadian proteins (PER1 and PER2) are downstream circadian proteins, and their level is tightly regulated by BMAL1. PERs proteins are vital for regulating many cellular functions, mainly cell proliferation and differentiation, regeneration, and transcription control and repression. As the levels of PER proteins decrease, BMAL1 accumulates due to reduced negative feedback, which facilitates the continuation of the circadian feedback loop [20]. We investigated the level of PER1 and PER2 in the serum of study samples. Our results revealed a significant decrease in both PER1 and PER2 levels in the vaping group compared to the non-vaping control group (Fig. 2A, B). Subsequently, we examined the level of PER1 and PER2 in the cardiovascular non-vaping group. The findings demonstrated a significant increase in PER1 and no significant change in PER2 in the cardiovascular non-vaping group compared to the values detected in the control group (Fig. 2A, B). The reduction in PERs proteins may explain the high level of BMAL1 in the vaping group, as it is considered the positive pole of the circadian feedback loop. These findings indicated that vaping elicited a significant disruption in the circadian system markers, which can negatively impact the physiological function in the body.

## The Effect of Vaping on Cryptochrome Circadian Proteins (CRY1 and CRY2)

**Fig. 3:**
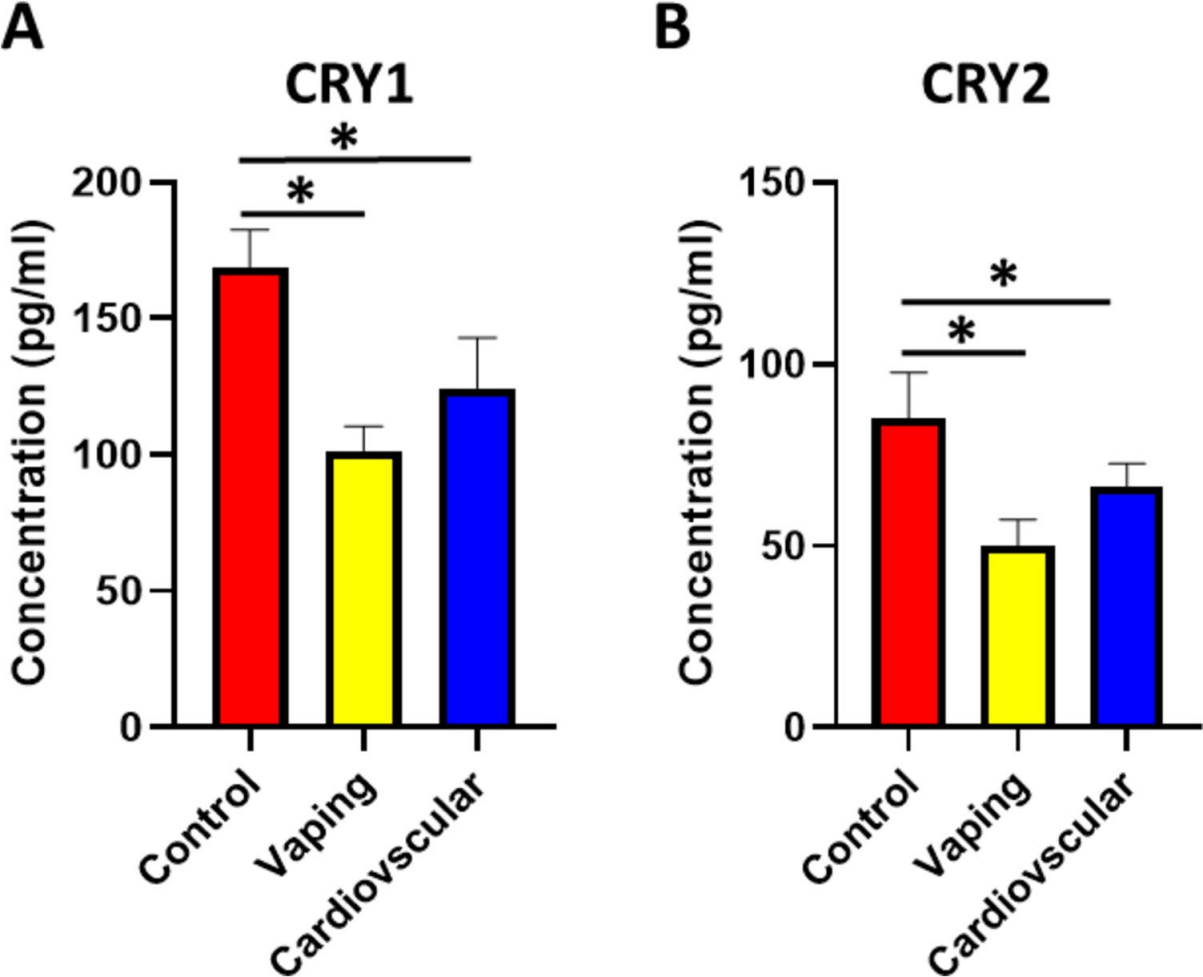
The serum levels of CRY1 and CRY2. **A** The serum CRY1 levels were significantly reduced in the vaping (F(2,251) = 9.87, p = 0.00008, η^2^ = 0.07, 95% CI [0.03, 0.12]; p < 0.001 vs. control) and cardiovascular (p = 0.001 vs. control) groups. **B** The serum CRY2 levels differed among groups (Kruskal–Wallis H(2) = 16.32, p < 0.001, r = 0.25), with lower levels in both vaping and cardiovascular groups compared to controls (*p* < 0.01). Data presented as mean ± SEM; *p < 0.05. The number of participants in the Control group, Vaping group, and Cardiovascular group are 90, 86,78 respectively

Cryptochromes (CRY1 and CRY2) are essential for balancing the circadian feedback loop by acting as the negative pole for suppressing the synthesis of upstream circadian markers; BMAL1 and preventing their excessive level. CRYs exert important regulatory roles for various physiological processes, including, hormone release, DNA repair, and metabolism [21]. We investigated the levels of CRY1 and CRY2 in the serum of study samples. The results indicated a significant decrease in both CRY1 and CRY2 levels in the vaping group compared to the non-vaping control group (Fig. 3A, B). Interestingly, our results also showed a significant decrease in CRY1 and CRY2 in the cardiovascular non-vaping group, which is consistent with the pattern observed in the vaping group. The low level of CRYs in vaping group disorganizes the circadian feedback loop and may explain the unchanged, high level of BMAL1 in the vaping group. These findings emphasize the potential adverse effects of vaping as it is considered a negative modulator for the circadian system and can ultimately increase the risk of developing heart problems.

## The Effect of Vaping on the Level of Oxidative Stress and Inflammation

Circadian system markers are reported to control the level of inflammation and oxidative stress in the human body [22]. The disturbance in circadian system markers observed in the vaping group prompted us to examine the level of inflammation and oxidative stress in vaping samples by measuring the levels of interferon gamma (IFN-γ), a cytokine involved in the inflammatory response, and malondialdehyde (MDA), a marker of lipid peroxidation and oxidative stress.

**Fig. 4:**
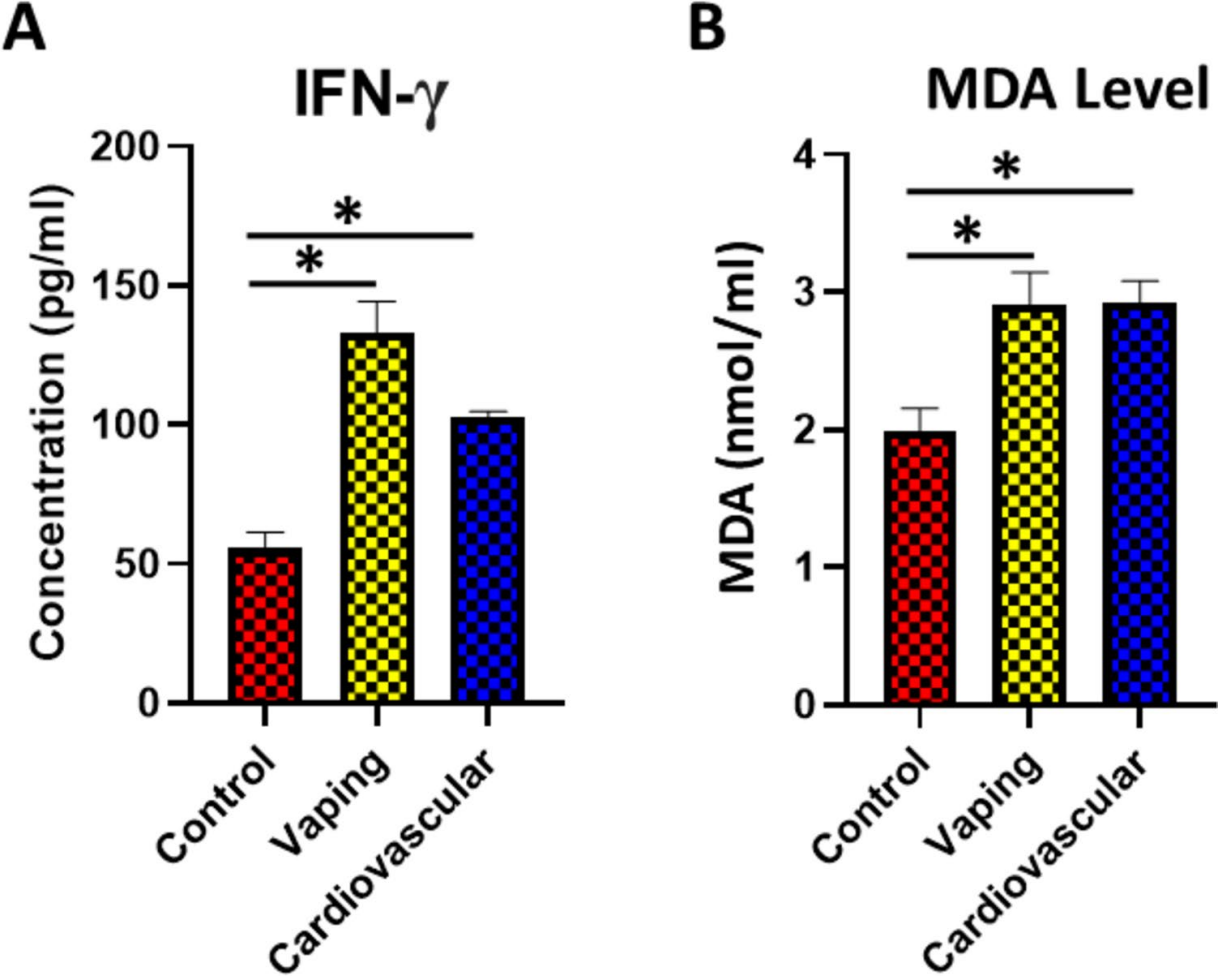
The serum levels of IFN-γ and malondialdehyde (MDA). **A** The serum IFN-γ levels were elevated in both vaping (F(2,251) = 14.05, *p* < 0.0001, η^2^ = 0.10, 95% CI [0.05, 0.15]; p < 0.01 vs. control) and cardiovascular (*p* < 0.01 vs. control) groups. **B** The serum MDA levels differed among groups (Welch’s ANOVA W(2,123.7) = 10.87, *p* < 0.001, η^2^ = 0.08, 95% CI [0.03, 0.13]); both vaping and cardiovascular groups had higher MDA than controls (Games–Howell post hoc *p* < 0.01). Data presented as mean ± SEM; *p < 0.05. The number of participants in the Control group, Vaping group, and Cardiovascular group are 90, 86,78 respectively

The results revealed a significant elevation in both IFN-γ and MDA levels in the vaping group compared to the non-vaping control group. This upregulation in IFN-γ and MDA was also detected in the cardiovascular non-vaping group (Fig. 4A, B). These findings indicated that the disturbance in circadian markers associated with vaping triggered intense inflammatory and oxidative stress and can be strongly correlated with future cardiac problems.

## Correlations Among Biomarkers

To explore potential relationships among the measured biomarkers, pairwise Spearman correlation coefficients were calculated between the six circadian proteins (Melatonin, BMAL1, PER1, PER2, CRY1, CRY2) and the inflammatory/oxidative stress markers (IFN-γ, MDA). Significant positive correlations were observed between Melatonin and PER1 (r = 0.42, p < 0.001) and CRY1 (r = 0.38, p = 0.01), while IFN-γ showed an inverse correlation with CRY2 (r = –0.35, p = 0.02). These results indicate that disruptions in circadian protein levels are associated with elevated inflammatory and oxidative stress markers, highlighting the interconnected effects of vaping on circadian regulation and cardiovascular risk.

## Discussion

Vaping has emerged as a safe and appealing alternative to traditional smoking cigarettes. It is widely used by younger adults and teenagers [23]. Unlike traditional cigarettes, which burn tobacco to produce smoke, vaping involves heating a liquid that contains nicotine mixed with flavoring spices and other chemicals and then the aerosol is inhaled [24]. The rapid rise in vaping has raised significant public health concerns, as its risks remain incompletely studied and poorly understood. Emerging evidence suggests that vaping may have extensive negative health effects on various body systems [25, 26]. The circadian system is an endogenous system that regulates various physiological functions in the human body mediated by upstream and downstream circadian-related proteins in response to local environmental cues, mainly the daylight [27]. The main role of this system is to control the 24-h biological clock [27]. The regulation of the internal molecular clock is found to profoundly impact many cellular functions, and it is crucial to ensure cellular homeostasis [10]. As vaping has increased rapidly, particularly among the young population, we opted to investigate the impact of vaping on various circadian system parameters and correlate the results with the possibility of increasing the risk of heart problems among vaping users.

Melatonin, often called the "sleep hormone," is produced by the pineal gland and helps signal the body to prepare for sleep, regulating sleep onset and quality [28]. It is considered the upstream inciter for the level of other circadian–related proteins, including BMAL1. Melatonin also has roles independent of initiating the circadian rhythm [28]. It has antioxidant properties and immune regulatory functions for helping the body to defend against cellular damage and infections [29]. Additionally, melatonin influences mood, reproductive health, and metabolism [30]. Disruptions in melatonin are reported to have downstream negative effects on cardiovascular functions [31]. Melatonin acts as a scavenger for reactive oxygen species (ROS), which is beneficial to curb the myocardial damage associated with ischemia–reperfusion injury [32]. Further, melatonin can improve the vagal tone of the heart and control heart rate and arterial pressure [33]. BMAL1 is a key component of the molecular clock system that regulates circadian rhythms in the human body [19]. By acting as a transcription factor, BMAL1 forms a complex with another protein called CLOCK to drive the level of various clock-controlled proteins, thus it is considered the positive pole of the circadian feedback loop [34]. Beyond its role in circadian rhythm regulation, BMAL1 also regulates various cellular processes, including cell proliferation, DNA repair, and regeneration [35]. BMAL1 is crucial for marinating the contractile functions of the heart by regulating the myocardial metabolic pathways [36]. It has been reported that deleting BMAL1 can instigate many remodeling changes in the heart and promote pressure overload [36]. Our study indicated that vaping reduced the level of melatonin and did not change the level of BMAL1. The reduction in melatonin seen in vaping users was similar to that shown in cardiovascular non-vaping patients indicating that vaping may increase the risk of heart disease. These findings raise concerns that vaping could increase the risk of heart problems by decreasing melatonin and omitting its heart-protective role.

PER1 and PER2 are downstream circadian-related proteins that are tightly regulated by BMAL1 [10]. The PERs are central to the "core" machinery of the circadian system, regulating the timing of various biological processes in alignment with the 24-h day-night cycle [37]. PERs are integral for ensuring healthy cardiovascular functions and preventing excessive cardiac damage during injury [38]. The cardiac–related functions of PERs are mediated by regulating cardiac fatty acid metabolism and preventing intense inflammatory response [39]. Our results showed that vaping significantly reduced the level of PER1 and PER2 while the level of both proteins was not changed in cardiovascular non-vaping patients indicating that vaping produces profound disruption in the circadian markers. Another significant set of circadian parameters examined includes the CRY1 and CRY2 proteins. CRYs act as the negative pole of the circadian feedback loop to repress the level of BMAL1 and other clock-related proteins to ensure proper synchronization of circadian rhythms [40]. CRYs also control various biological functions, such as metabolism and gene level. CRYs are essential for regulating cardiac metabolism, triglycerides, oxidative stress levels, and blood pressure [41]. The levels of CRY1 and CRY2 in vaping participants and cardiovascular non-vaping patients were remarkably reduced. The reduction in CRYs disrupted the negative pole of the circadian feedback loop and explained the unchanged and high level of BMAL1 reported in vaping users [19]. As circadian–related proteins control the level of systemic inflammation and oxidative stress and prevent their harmful elevation, we studied the level of inflammation and oxidative stress in vaping users. Our study revealed that the disruption in the circadian markers was correlated with marked upregulation in lipid peroxidation and INF-γ, which can negatively induce adverse effects in various cells, including cardiac cells. The observation that the levels of both IFN-γ and MDA in vaping users were notably comparable to those seen in cardiovascular non-vaping patients suggests that vaping may be associated with substantial inflammatory and oxidative stress responses in the heart, potentially increasing the risk of cardiovascular events over time. Although vaping is often perceived as a less harmful alternative to smoking, these findings indicate that it may still have significant adverse effects on the body system, especially the cardiovascular system. Vaping appears to disrupt the circadian system and induce imbalances in inflammatory and oxidative stress pathways [42]. Recent evidence supports our findings that vaping induces inflammatory and oxidative pathways similar to cardiovascular pathology [25, 26, 43]. These mechanisms involve immune activation and mitochondrial dysfunction, reinforcing our hypothesis of a potential association between vaping and increased cardiovascular risk. These clinically important results highlighted the need to plan future research to validate these results on larger population samples and correlate the results with other diseases. Further, the findings of our study implicate the necessity to implement new strategies to encourage young people to quit vaping by highlighting their unfavorable, long-term outcomes, particularly in disrupting the circadian system and inducing systemic inflammatory and oxidative stress responses that can worsen heart function.

## Study Limitations

Although our study provides important insights into the impact of E-cigarette use on circadian and inflammatory markers; several limitations should be acknowledged. First, this is a cross-sectional study which may limit the ability to infer causality between vaping and the observed molecular alterations. Longitudinal studies are necessary to validate whether these changes lead to long-term physiological dysfunctions that increase the risk of cardiovascular diseases. Second, while we controlled several confounding variables, there are several unmeasured factors, such as diet, physical activity, body mass index, and environmental exposures, that may influence the results. Third, as the vaping and the medical history are self-reported, there is a high chance of recall or reporting bias. Fourth, the circadian markers were measured at a single time point, which may not fully capture their diurnal variations. Fifth, correlation analyses were not performed due to limited data on BMI and other lifestyle variables. Time-series sampling and a larger population may be used in future research to validate and extend these findings. Finally, the incomplete covariate data (including BMI, physical activity, and dietary information) precluded formal adjustment for potential confounders in our analyses. Consequently, we cannot fully rule out the influence of these factors on the observed group differences.

## Conclusion

Our study revealed a novel correlation between vaping and the possibility of developing heart diseases mediated by disturbing the endogenous circadian system regulators. As the level of circadian-related markers decreased, regulatory control on inflammatory and oxidative stress was lost. These findings underscore the unexpected safety concerns related to vaping, as it can cause significant harm to the circadian system and impact its protective role on various body systems, including the cardiovascular system.

## Data Availability

The data that support the findings in this study are available from the corresponding authors upon reasonable request.

## Declarations

### Clinical Relevance

Studying the impact of E-cigarette on the circadian proteins helps raise awareness about the dark side of E-cigarette as it can disturb critical biological systems in our body. These deleterious mechanisms must be circulated to the public in order to make them understand that using E-cigarette is not entirely a safe option to conventional tobacco cigarettes and preventive measures must be planned to help youths and adult in quitting E-cigarettes.
